# Exploring the Pressure Characteristics of the PRESERFLO MicroShunt in In Vitro Studies and Effects of Sclera on Device Performance

**DOI:** 10.3390/jcm12237266

**Published:** 2023-11-23

**Authors:** Andi Masdipa, Sachiko Kaidzu, Masaki Tanito

**Affiliations:** Department of Ophthalmology, Shimane University Faculty of Medicine, Izumo 693-8501, Japan; m209402@med.shimane-u.ac.jp (A.M.); kecha@med.shimane-u.ac.jp (S.K.)

**Keywords:** glaucoma, PRESERFLO MicroShunt, in vitro studies, pressure characteristics, sclera, MIGS, intraocular pressure (IOP)

## Abstract

This study aims to investigate the pressure characteristics of the PRESERFLO MicroShunt, a microinvasive glaucoma device, using an in vitro setup. Additionally, the study explores the impact of the scleral tissue surrounding the device on its pressure and lumen area. Ten PRESERFLO MicroShunts were subjected to an in vitro experimental setup. A constant flow of physiological saline was maintained at 2 μL/min using an infusion syringe pump. The PRESERFLO was connected to a pressure transducer via a 23 G needle. Pressure characteristics were measured under three different conditions: without sclera [sclera (-)], passing through sclera at a 90° angle (sclera 90°), and passing through sclera at a 30° angle (sclera 30°). The lumen area of the device was measured using microscopic observation. We observed peak and trough pressures in this experimental setting; the peak pressure (6.76 mmHg) was significantly higher than the trough pressure of 4.74 mmHg (*p* = 0.0020) in the sclera (-) condition. Compared to sclera (-), the peak pressures were significantly higher in the sclera 90° (7.81 mmHg, *p* = 0.0020) and the sclera 30° (7.96 mmHg, *p* = 0.0039) conditions. Additionally, compared to sclera (-), the trough pressure was significantly higher in the sclera 90° (6.25 mmHg, *p* = 0.0039) and the sclera 30° (5.76 mmHg, *p* = 0.037) conditions. The lumen area was significantly smaller in the sclera 90° condition (3515 μm^2^) than the sclera (-) condition (3927 μm^2^, *p* = 0.0078). The study found that when the distal end of PRESERFLO MicroShunt was free and in air, it exhibited both peak and trough pressures. The presence of scleral tissue surrounding the PRESERFLO MicroShunt affects its lumen area and pressure characteristics. Understanding these effects can provide valuable insights into the device’s performance.

## 1. Introduction

Elevated intraocular pressure (IOP) is a risk factor for the development and progression of glaucoma [1,2]. Traditional surgical interventions for glaucoma, such as trabeculectomy and glaucoma drainage devices, are associated with notable complications and limitations [3,4]. In recent years, minimally invasive glaucoma surgery (MIGS) has emerged as a promising alternative for glaucoma treatment [5,6]. MIGS utilizes small devices and minimally invasive techniques to lower IOP, offering fewer complications and faster recovery [4,6,7].

The PRESERFLO MicroShunt, previously known as the Infocus MicroShunt, is a MIGS device made from a biocompatible and highly flexible polymer called styrene-block-isobutylene-block-styrene (SIBS) [8,9,10]. SIBS has been used in various medical applications, including coronary stents [11], and offers advantages such as greater biocompatibility and reduced risk of erosion and infection. PRESERFLO presents a promising option for glaucoma management [10,11].

The PRESERFLO, implanted in situ, is threaded through a knife followed by a needle tract under the limbus, connecting the anterior chamber to the space formed under the conjunctiva and Tenon’s capsule. Aqueous humor flows through the PRESERFLO and fills this space to form a bleb. While several publications have reported clinical outcomes and complications related to the PRESERFLO [12], there have been limited in vitro studies on this microshunt [13]. This study aims to measure the pressure characteristics of the PRESERFLO using an infusion pump system and to investigate the effect of porcine eye sclera on the device’s pressure and lumen area. The question studied in this article is whether there is a difference in pressure across the PRESERFLO and whether there is a change in lumen diameter if the PRESERFLO is passed through the scleral tissue. The pressure is measured ex vivo in sclera sections explanted from porcine eyes using a pressure transducer in line with a constant flow syringe pump infusion system.

## 2. Materials and Methods

### 2.1. Materials

**Figure 1:**
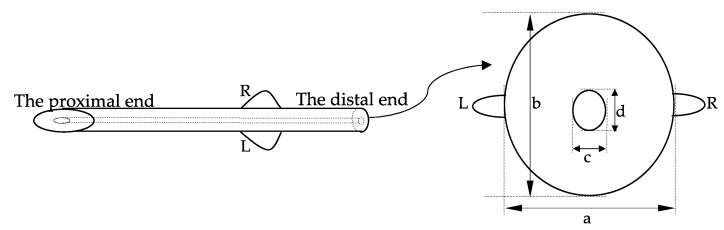
PRESERFLO specification. R, right; L, Left; a, horizontal outer diameter; b, vertical outer diameter; c, horizontal lumen diameter; d, vertical lumen diameter.

A total of ten PRESERFLO MicroShunts (Santen Pharmaceutical Co., Osaka, Japan) (Figure 1) provided by the company were used in this experiment. The specifications of PRESERFLO are shown in Figure 1. Physiological saline (0.9% sodium chloride) and disposable 23-G needles were purchased from NIPRO CORPORATION (Osaka, Japan). Syringes with 1 mL capacities were purchased from Terumo Corporation (Tokyo, Japan). Infusion tubes (JV-NDH1050FL and JV-ND1010PC) were purchased from JMS Co., Ltd. (Tokyo, Japan). An infusion syringe pump (SP101i) was purchased from Kd Scientific (Holliston, MA, USA). A pressure transducer (BLPR2), 4-channel transducer amplifier (SYS TBM4M), analog-to-digital converter (LAB-TRAX-4/16), and pressure curve analysis software LabScribe2 (LAB-TRAX-4) were purchased from World Precision Instrument (Sarasota, FL, USA). To insert PRESERFLO into the sclera, a specialized 0.5 mm and 1.0 mm double-step slit knife (MANI, Inc. Tochigi, Japan) was used to make an incision in the sclera. The porcine eyes used in this study were obtained from slaughterhouses on the day of the experiment and transported to the laboratory on ice within eight hours or less after the enucleation to prevent tissue degeneration. The use of these eyes in experiments does not fall under animal welfare regulations related to experiments using animals. The sclera was then separated from other tissues such as the cornea, uvea, retina, aqueous humor, and vitreous, then was rinsed with physiological saline.

### 2.2. Experimental Setting

**Figure 2:**
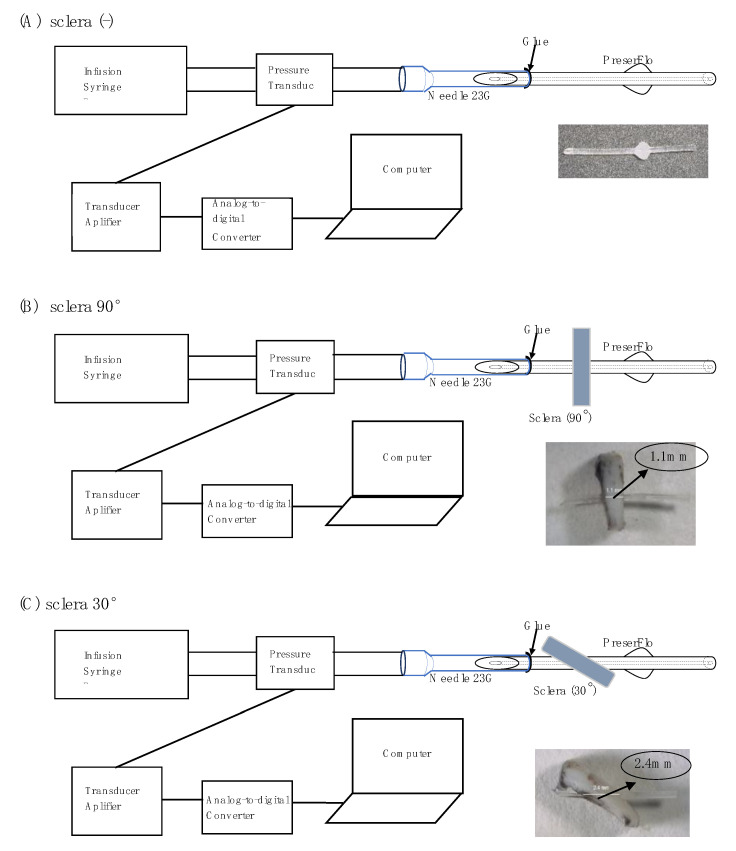
Configuration of pressure measurements in three different conditions: sclera (-) (**A**), sclera 90° (**B**), and sclera 30° (**C**).

The fundamental part of the pressure measurement system was the same as that in our previous report [14]. Figure 2 shows the schema of the experimental setup. A 1 mL syringe was attached to the infusion syringe pump to facilitate the flow of 2 μL/min of physiological saline to the pressure transducer through an infusion tube. From the pressure transducer, physiological saline flowed into a 23 G needle previously inserted with the proximal end of PRESERFLO (Figure 2). The pressure transducer was connected to a computer through the transducer amplifier and an analog-to-digital converter. Pressure characteristics were measured in three different conditions: sclera (-), sclera 90°, and sclera 30°. For the latter two conditions, a 5 × 5 mm full-thickness (1.1 mm thickness) scleral tissue sample was dissected from a porcine eye, and a 0.5 mm width scleral tunnel was created using a slit knife at an angle of either 90° (tunnel length of 1.1 mm) or 30° (2.2 mm). The tube was then passed through the scleral pieces. Figure 2A–C show the sclera (-), sclera 90°, and sclera 30° conditions, respectively.

The beveled tip at the proximal end of the PRESERFLO was inserted into a 23 G needle end with a length of 1.5–2 mm. The space between the 350 µm outer diameter of the PRESERFLO and the 420 µm inner wall of the 23 G needle was filled with glue (TOMBOW PENCIL CO., Ltd., Aichi, Japan) to prevent peri-annular leakage, and the glue was allowed to dry for at least 10 min. After applying the glue, a waiting time of about 10 min was allowed, followed by a flush test using physiological saline to ensure there were no leaks. The needle and PRESERFLO were then flushed with physiological saline to assure no leakage and to assure that the lumen was patent.

Once all the devices were connected as shown in Figure 2 and the absence of trapped air in the tube was confirmed, the transducer amplifier was calibrated by waiting until the pressure graph showed a constant value. A flow of 2 µL/min was started until the pressure reached a peak, which was defined as the peak pressure (Figure 3①). At that time, droplets began to form at the distal end of the PRESERFLO. Subsequently, the pressure decreased, and this was accompanied by an increase in droplet size. The droplet size continued to grow until the droplet fell, and at that point, the pressure showed the lowest value, defined as the trough pressure (Figure 3②). In each PRESERFLO device, the peak and trough pressures were recorded three times for each of the three different conditions shown in Figure 2 (a total of 9 recordings for each device). In each condition, the average value of the three recordings was calculated for each device.

The appearance of the distal end of the PRESERFLO in the sclera (-) (Figure 4A) and sclera 90° (Figure 4B) conditions was obtained using a multi-angle stereo microscope and digital camera system (VB-7010/VB-G25, Keyence Co. Ltd., Osaka, Japan) with a magnification of 175×. The horizontal diameter (Figure 1c) and vertical diameter (Figure 1d) of the PRESERFLO lumen in the obtained images were measured using the scale provided by the microscope application. The lumen area of the PRESERFLO was then calculated using Equation (1):

(1) $$A=\pi \times \frac{c}{2}\times \frac{d}{2}$$

where *A* is the lumen area, *c* is the horizontal diameter, and *d* is the vertical diameter.

### 2.3. Data Analysis

The data analysis was performed using JMP Pro 16 statistical software (JMP Statistical Discovery, Cary, NC, USA). All data are presented as the median value and interquartile range (IQR). Paired comparisons were conducted using the Wilcoxon signed-rank test.

## 3. Results

The peak and trough pressures were recorded for 10 PRESERFLO devices. Based on the observation, the peak pressure was consistently recorded when droplets began to form at the distal end of the device, and the trough pressure was consistently recorded when the formed droplets fell from the distal end of the device. The measured pressure values are summarized in Table 1.

In the sclera (-) condition, the peak pressure of 6.76 mmHg was significantly higher than the trough pressure of 4.74 mmHg (*p* = 0.0020). Similarly, in both the sclera 30° and sclera 90° conditions, the peak pressures were significantly higher than the trough pressures.

When comparing the different conditions, the peak pressure was significantly higher in the sclera 90° condition (7.81 mmHg, *p* = 0.0020) and the sclera 30° condition (7.96 mmHg, *p* = 0.0039) compared to the sclera (-) condition. Additionally, the trough pressure was significantly higher in the sclera 90° condition (6.25 mmHg, *p* = 0.0039) and the sclera 30° condition (5.76 mmHg, *p* = 0.037) compared to the sclera (-) condition. However, there was no significant difference in either the peak or trough pressures between the sclera 90° and 30° conditions (*p* > 0.05).

The lumen area was measured for eight PRESERFLO devices under both the sclera (-) and sclera 90° conditions (Table 2). The lumen area in the sclera (-) condition was found to be 3927 μm^2^. In contrast, the lumen area in the sclera 90° condition was significantly narrower, measuring 3515 μm^2^ (*p* = 0.0078).

## 4. Discussion

In this study, we evaluated two pressure characteristics of the PRESERFLO using an infusion pump system. Two consistently observed pressures, namely, peak and trough pressures, were recorded. Based on our observations, the presence of scleral tissue around the device was associated with an increase in both peak and trough pressures, as well as a decrease in the lumen area of the device.

The phenomenon of peak pressure occurred when physiological saline began to flow from a steady state or shortly after the formed droplets fell. At this point in time, the distal end of the PRESERFLO was not completely filled with physiological saline. Considering the hydrophobic nature of the SIBS material and the fluid dynamics at play, including drag force induced by viscosity and capillary forces resulting from surface tension, a higher pressure was necessary in order to expel the physiological saline from the tube (Figure 5).

The pressure increase caused by drag and capillary forces can be described using Equation (2) [14]:

(2) $$\Delta P=\frac{8\eta xv}{r^{2}}-\frac{2\gamma \cos\theta }{r}$$

where Δ*P* is the pressure difference, 𝜂 is the fluid viscosity, 𝛾 is the surface tension, 𝑣 is the fluid velocity, x is the distance between the contact angle and the tip of the tube, 𝑟 is the inner radius of the tube, and 𝜃 is the contact angle [14]. Referring to Equation (2) and Figure 5, it is evident that the variable that undergoes change is x.

**Figure 3:**
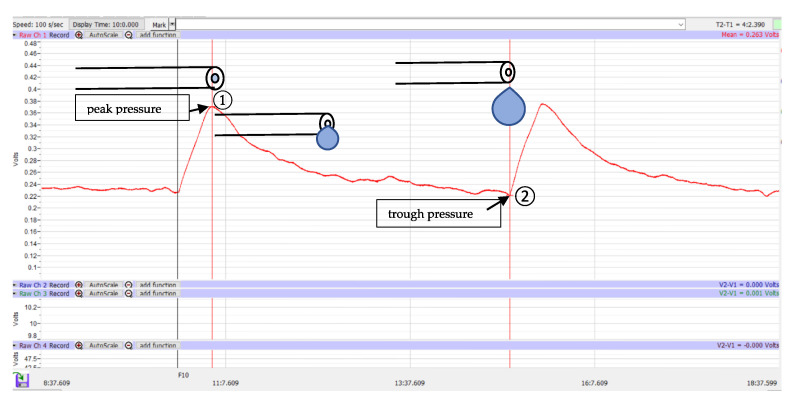
Graph of pressure analysis: peak pressure (**①**) and trough pressure (**②**).

The distal end of the PRESERFLO remained free from any attachments to tissues or other objects in the experimental settings. Due to the hydrophobic properties of SIBS, droplets emerged in spherical shapes and gradually grew in size without immediate detachment. The cohesive forces between the liquid particles generated a surface tension that enabled the droplets to maintain their spherical shapes and prevented them from wetting the exterior of the PRESERFLO tube. These cohesive forces were strong enough to support the weight of the droplets until they reached a critical size and eventually detached. The size of the formed droplets correlated with their weight, which in turn affected the resultant force at the distal end of the PRESERFLO. As a result, the measured pressure decreased (Figure 3). The droplets fell when their weight exceeded the attractive force between the liquid particles [15], a point that is also evident on the graph where the trough pressure (Figure 3②) was recorded. From these phenomena, in an actual clinical situation where the PRESERFLO is implanted into the eye, it is anticipated that only the peak pressure is observed when the distal end of the PRESERFLO is contacted with water.

By equating the attractive force $\left(F_{ST}\right)$ of the water particles to the weight (w) of the formed droplet, the radius of the droplet (*r*) can be calculated using the following equations:

(3) $$w=F_{ST}$$

(4) $$w=m\times g=(\rho \times \left(\frac{4}{3}\pi r^{3}\right))\times g$$

(5) $$F_{ST}=\gamma \times L\;$$

(6) $$L=2\pi r_{p}=\pi D$$

Here, L represents the circumference of the PRESERFLO lumen, ρ is the density of water, and γ is the surface tension. Substituting Equations (4) and (5) into Equation (3) results in Equation (6).

Solving for *r* yields:

(7) $$\rho \times (\frac{4}{3}\pi r^{3})\times g=\gamma \left(\pi D\right)$$

(8) $$r=\sqrt[{3}]{\frac{3\gamma D}{4\rho g}}=\sqrt[{3}]{\frac{3\times \left(72\times 10^{-3}\right)\times (7\times 10^{-5})}{4\times 1000\times 10}}=\sqrt[{3}]{378\times 10^{-12}}=0.723\times 10^{-3}m$$

The estimated diameter value (*D*) of $1.446\times 10^{-3}m$ should be four times larger than the external lumen diameter of PRESERFLO at 350 μm. This estimation aligns well with the actual experimental phenomenon (Figure 6).

After the generation of peak pressure and when the pressure stabilizes, the pressure resistance value can be calculated using the Hagen–Poiseuille equation. The pressure resistance attributed to the PRESERFLO device has been discussed in several previous publications employing the Hagen-Poiseuille equation [13].

(9) $$\Delta P=\frac{8\mu lQ}{\pi r^{4}}=\frac{8\pi \mu lQ}{A^{2}},\;A=\pi r^{2}$$

In this equation, ∆*P* (i.e., *P*_1_ − *P*_2_) represents the pressure difference between the two ends, *l* is the length of the pipe (8.5 mm = 8.5^−3^ m), *μ* is the dynamic viscosity (10^−3^ Pa·s), *Q* is the volumetric flow rate (2 μL/min = $\frac{1}{3}\times 10^{-12}m^{3}\cdot s^{-1})$, and *A* is the cross-sectional area of the lumen. This equation allows for the calculation of pressure resistance and provides insight into the behavior of the PRESERFLO device.

**Figure 4:**
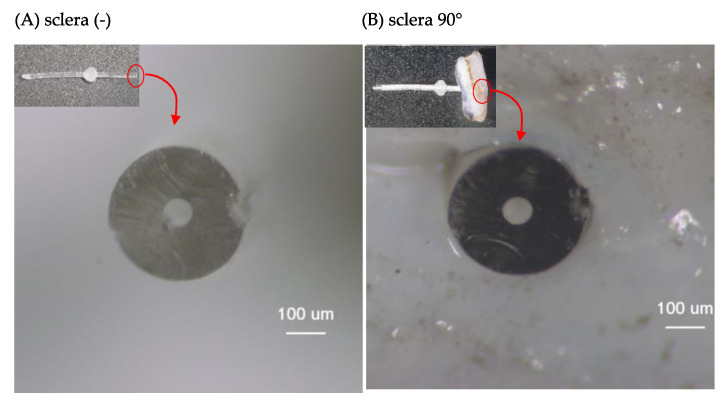
Cross-sectional images of the distal end of the PRESERFLO under different conditions: (**A**) sclera (-) and (**B**) sclera 90°.

**Figure 5:**
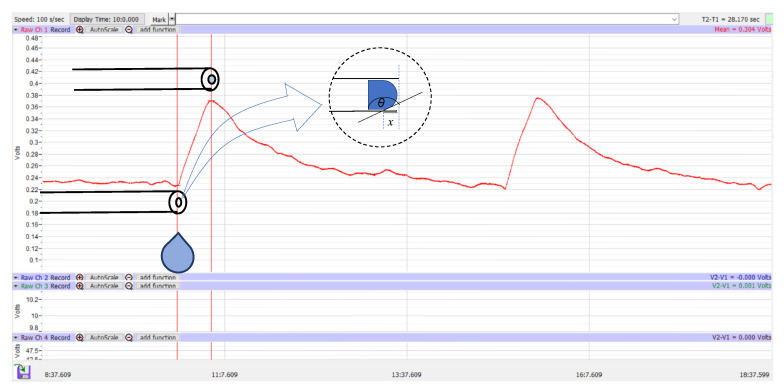
A graph illustrating the peak pressure occurring with the liquid at the end of the tube.

**Figure 6:**
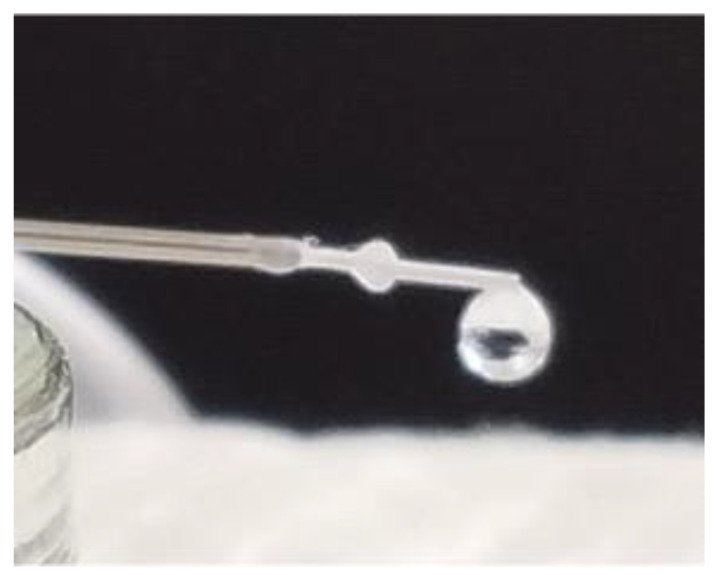
An image of spherical droplets formed at the distal end of PRESERFLO during the experiments (with sclera (-) condition).

**Figure 7:**
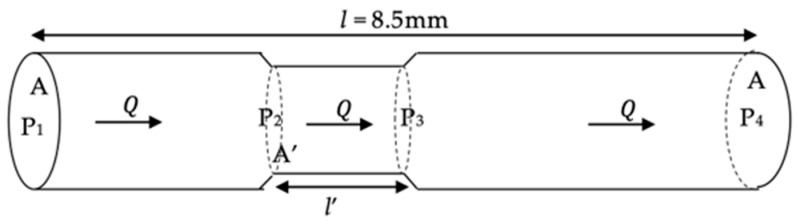
An illustration of a PRESERFLO tube with a change in the lumen area as it passes through the sclera.

The Hagen–Poiseuille Equation (9) is typically applicable when the cross-sectional area along the lumen remains constant. However, in this study, variations occurred in the cross-sectional area of the PRESERFLO lumen as it traversed the sclera (Figure 4). Additionally, changes in the angle between the sclera and PRESERFLO led to alterations in the length of the tube passing through the sclera (Figure 2B,C). These fluctuations in area (*A*) and length (*l*) were two factors impacting the pressure resistance value in the Hagen–Poiseuille equation. As a consequence of these variations in variables (Figure 7), the Hagen–Poiseuille equation was transformed into the following form:

(10) $$\Delta P=\Delta P_{12}+\Delta P_{23}+\Delta P_{34}$$

(11) $$\Delta P=8\pi \mu Q\left(\frac{l_{1}}{A^{2}}\right)+8\pi \mu Q\left(\frac{l^{\prime }}{{A^{\prime }}^{2}}\right)+8\pi \mu Q\left(\frac{l_{2}}{A^{2}}\right)$$

(12) $$l=l_{1}+l_{2}+l^{\prime }$$

(13) $$\Delta P=8\pi \mu Q\left(\frac{l-l^{\prime }}{A^{2}}+\frac{l^{\prime }}{{A^{\prime }}^{2}}\right)$$

In this modified equation, changes in both the length and cross-sectional area are accounted for, providing a more accurate representation of the pressure resistance in the context of the experimental setup.

By substituting the relevant values for each variable into Equation (9), the pressure resistance for the condition without sclera (Δ*P*_(-)_) was calculated as 3.19 mmHg. Utilizing the lumen areas (*A* and *A*′) obtained from our experiments (Table 2), and by applying Equation (13), the pressure resistance values for the conditions with sclera at 90° (Δ*P*_90_) and 30° (Δ*P*_30_) were determined to be 3.62 mmHg and 3.75 mmHg, respectively. These mathematical calculations of pressure resistance using the Hagen–Poiseuille equation corresponded with the results presented in Table 1, demonstrating that the pressures exhibited by the PRESERFLO when passing through the sclera were higher compared to the condition without sclera.

Through theoretical calculations, the differences in Δ*P* between the condition without sclera and the sclera at 90° and 30° relative to the condition without sclera were determined to be 0.43 mmHg (3.62 mmHg–3.19 mmHg) and 0.56 mmHg (3.75 mmHg–3.19 mmHg), respectively. In the actual data, presented in Table 1, the difference in peak pressure was observed to be 1.05 mmHg (7.81 mmHg–6.76 mmHg) between the condition without sclera and the sclera at 90°, and 1.20 mmHg (7.96 mmHg–6.76 mmHg) between the condition without sclera and the sclera at 30°. Conceptually, the difference in ∆*P* (i.e., ∆*P*_90_ − ∆*P*(-) or ∆*P*_30_ − ∆*P*_(-)_) should equate to the same value as the difference in peak pressure (*P*_1_) (i.e., *P*_1 90_ − *P*_1(-)_ or *P*_1 30_ − *P*_1(-)_) among the three different conditions (condition without sclera, sclera at 90°, and sclera at 30°). Thus, the disparities between the ∆*P* differences derived from the Hagen–Poiseuille equation and the experimentally-derived *P*_1_ differences suggest the presence of unidentified factors that influence PRESERFLO resistance.

In the aforementioned equation, ΔP signifies the pressure difference between the distal and proximal ends of the PRESERFLO. The pressure at the proximal end (*P*_1_) should represent the IOP of a human eye implanted with a PRESERFLO device. Unlike our experimental setup, when the PRESERFLO device is implanted in a human patient’s eye, the distal end of the PRESERFLO is not free [16,17], and the resistance attributed to Tenon/subconjunctival tissues comes into play. Consequently, after surgical implantation of the PRESERFLO device, the IOP is expected to be higher than the peak pressure observed in the sclera 30° condition in our study. Clinical studies have shown that the post-surgical IOP in eyes with PRESERFLO implants is indeed greater than the peak pressures reported in our study. In previous research, baseline IOP values decreased from their initial levels after PRESERFLO implantation. In 23 eyes, the baseline IOP of 23.8 ± 5.3 mmHg decreased to 10.7 ± 3.5 mmHg at 3 years and to 12.4 ± 6.5 mmHg at 5 years post-implantation [17]. In 164 eyes, the baseline median IOP of 20 mmHg (IQR 16.5–26) decreased to 12 mmHg (IQR 10–15) at 1 year [18]. In 395 eyes, the baseline IOP of 21.1 ± 4.9 mmHg decreased to 14.3 ± 4.3 mmHg at 1 year [19]. These clinical findings align with expectations, as the reported post-surgical IOP values were higher than the peak pressures observed in our study. One common early complication following PRESERFLO implantation is hypotony, defined as an IOP below 6 mmHg [16,20,21]. Hypotony can occur if there is an excessive flow of aqueous humor through the gap between the tube and the sclera, especially if the scleral tunnel width is too wide. Reduced flow rates could also lead to decreased tube resistance and subsequent hypotony. Previous measures, such as placing removable polyamide sutures inside the PRESERFLO lumen, have effectively reduced the incidence of post-surgical hypotony [16]. The mechanisms underlying hypotony through reduced inflow and an increase in tube resistance by narrowing the lumen are well-explained by the equations discussed in this study. These equations provide insight into the factors influencing IOP regulation and the potential complications associated with PRESERFLO implantation.

It is crucial to acknowledge that this study was conducted in a controlled laboratory setting and has inherent limitations. The continuous flow of physiological saline and pressure measurements executed using laboratory equipment might not entirely replicate the dynamic conditions within a living organism. Additionally, the use of porcine scleral samples may introduce structural variations when compared to human sclera. Interestingly, a previous study reported a ΔP value of 2.6 mmHg for a flow rate of 2 µL/min in gravity-based flow settings for PRESERFLO [13]. The observed differences could potentially be attributed to the distinct effects of surface tension and hydrostatic pressure, providing a potential explanation for the variation in the measured PRESERFLO resistance between the prior and current studies. Despite these limitations, the findings of this study are expected to lay the groundwork for further investigations in this field. This research contributes valuable insights as to the pressure characteristics of the PRESERFLO device, although further in vivo research is necessary in order to validate these findings and to better understand the behavior of the device within living systems.

## 5. Conclusions

In this in vitro study, we have discovered that the PRESERFLO device, composed of SIBS material, exhibits distinctive pressure characteristics. The presence of a free distal tip without attachments led to the observation of peak and trough pressures. The inclusion of scleral tissue around the PRESERFLO tube resulted in a reduction in the tube’s lumen area. Moreover, variations in the angle formed between the PRESERFLO device and the sclera brought about changes in the variables present in the Hagen–Poiseuille equation, leading to an increase in pressure resistance. These findings shed light on the complex interplay between the PRESERFLO device, the surrounding tissue, and the resultant pressure dynamics. It underscores the importance of considering factors such as lumen area, material properties, and anatomical context when evaluating the behavior of such devices, providing valuable insights for both future research and potential clinical applications.

## Figures and Tables

**Table 1:** Comparison of peak and trough pressures among sclera (-), sclera 90°, and sclera 30° conditions (N = 10 in each group).

Pressure,	Median (IQR)			*p* Value		
mmHg	(-)	90°	30°	(-) vs. 90°	(-) vs. 30°	30° vs. 90°
Peak	6.76 (6.10–7.14)	7.81 (7.06–8.16)	7.96 (6.71–8.55)	0.0020	0.0039	0.77
Trough	4.74 (4.07–5.45)	6.25 (5.02–7.11)	5.76 (5.07–6.07)	0.0039	0.037	0.57
*p* value	0.0020	0.0020	0.0020			

*p* values were obtained using Wilcoxon signed rank. IQR, interquartile range.

**Table 2:** Comparison of the lumen’s cross-sectional area between sclera (-) and sclera 90° conditions (N = 8 in each group).

Area, μm^2^	Sclera (-)	Sclera 90°	*p* Value
Median (IQR)	3927 (3804–4108)	3515 (3342–3900)	0.0078

*p* value was obtained using the Wilcoxon signed rank. IQR, interquartile range.

## Data Availability

The data are fully available upon reasonable request to the corresponding author.
